# Heteroarotinoids with Anti-Cancer Activity Against Ovarian Cancer Cells

**DOI:** 10.2174/1874104500701010011

**Published:** 2007-10-24

**Authors:** Thanh C. Le, K. Darrell Berlin, Stacy D. Benson, Margaret A. Eastman, Gianna Bell-Eunice, Anna C. Nelson, Doris M. Benbrook

**Affiliations:** 1 Department of Chemistry, Oklahoma State University, Stillwater, OK 74078, USA; 2 Department of Chemistry, Southwestern Oklahoma State University, Weatherford, OK 73069, USA; 3 Departments of Obstetrics & Gynecology, Biochemistry and Molecular Biology, University of Oklahoma Health Sciences Center, 975 N.E. 10th St., Rm 1374, Oklahoma City, OK 73190, USA

## Abstract

The Flex-Het compound **10a** (SHetA2-NSC 721689) {[4-nitrophenylamino][(2,2,4,4-tetramethylthiochroman-6-yl)amino]methane-1-thione]} has shown promise in preclinical testing as an anti-cancer agent without evidence of toxicity, skin irritancy, or teratogenicity. One objective of this study was to synthesize a series of heteroarotinoids structurally related to SHetA2 and to measure the effect of structural alterations on the cytotoxicity activities of the compounds on A2780 ovarian cancer cells. Alterations included comparisons of activity of an NO2 end group versus a CO2Et end group, a thiourea linker versus a urea linker, and a distorted, thiochroman ring unit versus a planar quinoline ring unit. Cytotoxicity assays demonstrated the thiourea linker compounds to be similar in potency to the urea linker counterparts, the NO2 substitutions were slightly more potent than the CO2Et substitutions, and replacement of the thiochroman group with a planar quinoline fused ring system markedly reduced activity. The mechanism of cytotoxicity through apoptosis was confirmed for the compounds. The optimal combination of structural features for enhancing potency consisted of a urea linker, a NO2 substitution, and a flexible thiochroman unit. Extensive H-bonding in the more active urea derivative was confirmed by X-ray and NMR analyses. This is the first example in which the biological activity of flexible, thiochroman units is compared to that of fused aryl units in a heteroarotinoid molecule.

## INTRODUCTION

Retinoid biology and chemistry remains an active area in cancer chemotherapy with certain synthetic systems exhibiting reduced toxicity compared to natural retinoids [1-5]. Heteroarotinoids are a family of heterocycles which have demonstrated such reduced toxicity [6-7]. To illustrate, endogenous *trans*-retinoic acid (**1**) has a maximum tolerated dose (MTD) of 10 mg/kg/day in mice which is comparable to a dose of 9.4 mg/kg/day for **2** (X = O, R = H). In contrast, acids **3** possessed MTD values of 32 mg/kg/day and 34 mg/kg/day for X = O and X = S, respectively [7]. Since natural retinoids act with nuclear receptors to mediate biological activities by binding to specific DNA sequences [8-10], two members of **2** were examined and were selective for retinoic acid receptors (RARs) [7]. Recent studies have suggested that transcriptional activation of RARγ by some retinoids may correlate with toxicity [11-12]. Thus, RAR activation by synthetic retinoids may have limitations in terms of useful chemotherapy, although more study is needed. Two members of **3** (X = O, S) were good inducers of differentiation in human HL-60 cells (promyelocytic cells) with ED_50_ values at the nanomolar level and similar to that of **1** and better than the two members of **2** (X = O, R = H and Et) which had ED_50_ values in the micromolar range [13]. Thus, biological activity with such systems is sensitive to small structural changes.

Increasing the length and nature of the linker between the two aryl rings in **2** resulted in the generation of selected examples of **4**. The urea and thiourea linkers in **4** added a flexible property to the molecules which was reasoned initially to be better accommodated in the RARγ receptor [14-19]. In three examples of **4** examined to date [16], the agents, termed Flexible Heteroarotinoids (Flex-Hets) were receptor independent/apoptotic as diagnosed *via *the treatment of ovarian primary and cancerous cell lines OVCAR-3, Caov-3, and SK-OV-3. Later work revealed that the compounds inhibited multiple types of malignant cell lines and similar ovarian malignant cell lines with only very weak activity against normal and benign cells [17-19]. Growth inhibition was correlated with apoptosis and the generation of reactive oxygen species. Synthetic retinoids as inducers of apoptosis in a variety of carcinoma cells have been demonstrated, for example, with 6-[3-(1-adamantyl)]-4-hydroxylphenyl-2-naphthalenecarboxylic acid (**5**) without receptor activation [20-24]. Evidence for the molecular pathways for inducement of apoptosis in ovarian cancer cells by **5** and 4-HPR (**6**) was recently made available [20-23]. One facet of the mechanism of action was the depolarization of the mitochondrial membrane. The mechanism by which **4** induces apoptosis in cancer cells over normal cells however, has been shown to occur through direct effects on the mitochondria and Bcl-2 family of proteins [25]. Apoptosis is currently recognized as an avenue for cancer chemotherapy [21]. The current common line of treatment for ovarian cancer is the use of cisplatin (Platinol) or carboplatin (Paraplatin). However, resistance to cisplatin has become a major issue [26].

In an effort to further elucidate structural-activity relationships with systems containing the urea/thiourea linkers related to **4**, a series of heteroarotinoids was prepared with a large hydrophobic group as the fused-aryl ring unit and examined for ability to inhibit growth of A2780 ovarian cancer cells. In view of the biological response to conformationally restricted retinoids, such as TTNPB (**7**) which is toxic, however, and the clinical utility of Tazarotene (**8**), systems **9** were prepared with a nitrogen atom in a planar quinoline unit. For the sake of comparison of activities, members of **10** possessing a sulfur atom in a flexible, thiochroman ring unit were evaluated. A search of the literature did not reveal any heteroarotinoids with diheteroaryl-substituted, flexible linking urea or thiourea groups structurally similar to **9**. Diaryl-substituted retinoids with rigid linkers or void of linking groups between the rings are known, such as with 6-[3’-(1-adamantyl)-4’-hydroxyphenyl]-2-naphthalenecarboxylic acid (**5**), which possess ability to inhibit growth of cancerous cells and to induce apoptosis independent of receptor activation [24]. The recent example of a similar structure **11** [IC_50_ = 1.76 μM] possessed strong inhibitory ability against chloroquine-resistant strain of *Plasmodium falciparum*, the human malaria parasite. No specific mechanism of action was reported [27-29]. It is also noteworthy that the related heteroarotinoid **12** displayed moderate activity against the growth of *Mycobacterium bovis* BCG [30]. 

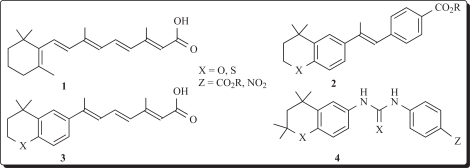

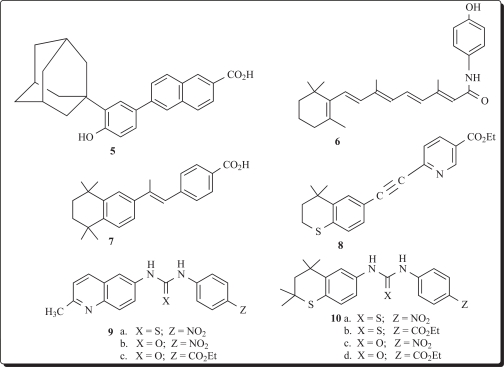

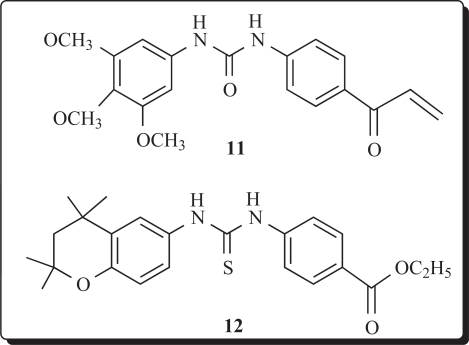







## MATERIALS AND METHODOLOGY

### General Chemistry Information

Compounds **10a**, **10b**, and **10d** were prepared from literature procedures [17-19].

In a typical experiment, the equipment used was a 25-mL, three-necked, round-bottomed flask equipped with a N_2_ inlet, a condenser, a magnetic stirrer and an addition funnel. Glassware was oven-dried prior to use and flushed with N_2_ when assembled. All isocyanates and isothiocyanates were obtained from Aldrich Chemical Company, Milwaukee, WI. Commercial solvents were used as received except all were dried over molecular sieves 3a prior to use. Extended periods of drying over molecular sieve 3a were required with THF to minimize the water content and maximize the yields of products from the condensation of the isocyanates (isothiocyanates) with the appropriate amines. Melting points of products were taken on a Thomas-Hoover capillary unit and were uncorrected. IR spectra were recorded on a Perkin-Elmer 2000 FT-IR as liquid films or as KBr pellets. The NMR spectra were taken on a Varian Unity Gemini 300 MHz unit operating at 300.082 MHz [^1^H] and at 75.463 MHz [^13^C]. Some spectra were taken on a Varian Unity INOVA 400 MHz unit operating at 399.99 MHz [^1^H] and at 100.01 MHz [^13^C]. Most samples were dissolved in DCCl_3_, unless otherwise specified, and signals were referenced to TMS [δ for ^1^H; ppm for ^13^C] at RT. The ^15^N NMR experiments [tuned to 60.65 MHz] were conducted in DMSO-*d*_6_ on a Varian Unity INOVA 600 MHz unit. Chemical shifts for ^15^N in samples were referenced to H_4_^15^N-^15^NO_2_ [^15^N 95% doubly enriched + 85% D_2_O + 5% H_2_O + 10% HNO_3_] and then to liquid ammonia. TLC analysis employed Polygram Polyester sheets (0.2 mm thick) and were UV sensitive [UV_254_]. A Waters Corporation semi-preparative HPLC unit [model 2487, dual λ detector, Sunfire prep silica column] was employed to determine purity of all samples in H_2_CCl_2_. Elemental analyses were performed by Atlantic Microlab, Inc., Norcross, GA 30091. 6-Amino-2-methylquinoline (**13**, 97%, mp 189-191°C) was purchased from Alfa Aesar Company, Ward Hill, MA 01835-6904, and was used directly.

### General Chemistry Procedures

As a general procedure, the mixture was cooled to 0°C (ice bath), and 4-nitrophenyl isocyanate (1.04 eq) (or 4-nitrophenyl isothiocyanate or other substituted derivatives) in dry THF (5 mL) was added dropwise (~5 min) to a solution of the required amine (1.00 eq) in dry THF (3 mL). After the addition, the reaction mixture was allowed to warm to RT and was then stirred (magnetic) for 24 h. During the 24 h period of stirring, additional THF was added to maintain volume. The solvent was removed *in vacuo* to give a heavy oil, which was crystallized, or a solid which was recrystallized from a suitable solvent or was dissolved in an appropriate solvent (see individual experiments for the solvent used) and subjected to flash chromatography [Baker silica gel, 40 μm particle size] to give a pure product.

#### 1-(2-Methylquinolin-6-yl)-3-(4-nitrophenyl) thiourea (9a).

Commercial 6-amino-2-methylquinoline (**13**, 158 mg, 1.0 mmol) dissolved in dry THF (6 mL) was cooled to 0°C (ice bath), and 4-nitrophenyl isothiocyanate (184 mg, 1.02 mmol) in dry THF (5 mL) was then added dropwise. After the addition, the reaction mixture was allowed to warm to RT and was then stirred for 24 h. The THF was then removed *in vacuo* to give a dark yellow solid. The solid was subjected to flash chromatography [EtOAc:hexanes,(1:2)] on silica gel and gave **9a** (268 mg, 79.2%) as a bright yellow solid: mp 113-115°C; IR (KBr) 3285 [NH], 3172 [NH] cm^-1^; ^1^H NMR (DMSO-*d*_6_) δ 2.63 (s, 3 H), 7.38 (d, 1 H, *J *= 8.6 Hz), 7.78 (d, 2 H, *J *= 9.2), 7.87 (m, 3 H, *J *= 8.6 Hz), 8.03 (s, 1 H) 8.22 (d, 2 H, *J* = 9.2), 9.75 (s, 1 H, NH), 10.24 (s, 1 H, NH); ^13^C NMR (DMSO-*d*_6_) ppm 24.7 [CH_3_], 120.3-158.1 [Ar-C], 179.5 [C=O]. Anal. (C_17_H_14_N_4_O_2_S) C, H, N, S: C, 60.34; H, 4.17; N, 16.56, S, 9.48. Found: C, 60.12; H, 4.20; N, 16.39; S, 9.44.

#### 1-(2-Methylquinolin-6-yl)-3-(4-nitrophenyl)urea (9b).

 6-Amino-2-methylquinoline (**13**, 158 mg, 1.0 mmol) dissolved in dry THF (6 mL) was cooled to 0°C (ice bath), and 4-nitrophenyl isocyanate (167 mg, 1.02 mmol) in dry THF (5 mL) was then added dropwise. The remaining procedure was the same as for **13a** and yielded a brownish yellow solid. The solid was subjected to flash chromatography [*i*-propanol: hexanes (1:1)] on silica gel and gave **9b** (201 mg, 62.3%) as a bright yellow solid: mp 241-242°C; IR (KBr) 3330 [NH], 3295 [NH] cm^-1^; ^1^H NMR (DMSO-*d*_6_) δ 2.60 (s, 3 H), 7.34 (d, 2 H, *J *= 8.6 Hz), 7.63 (m, 1 H), 7.71 (d, 1 H, *J *= 6.7 Hz), 7.85 (d, 2 H, *J *= 8.6 Hz), 8.14 (m, 1 H), 8.19 (d, 2 H, *J* = 6.7 Hz), 9.19 (s, 1 H, NH), 9.52 (s, 2 H, NH); ^13^C NMR (DMSO-*d*_6_) ppm 24.7 [CH_3_], 120.3-158.1 [Ar-C], 179.5 [C=O]. Anal. (C_17_H_14_N_4_O_3_S) C, H, N, S. C, 63.35; H, 4.38; N, 17.38. Found: C, 63.07; H, 4.44; N, 17.32.

#### Ethyl 4-[3-(2-Methylquinolin-6-yl)ureido]benzoate (9c).

 6-Amino-2-methylquinoline (**13**, 158 mg, 1.0 mmol) dissolved in dry THF (6 mL) was cooled to 0°C (ice bath), and 4-ethoxycarbonylphenyl isocyanate (195 mg, 1.02 mmol) in dry THF (5 mL) was then added dropwise. The remaining procedure was as for **9a** and led to a yellow solid. Recrystallization (methanol) gave **9c** (290 mg, 69.5%) as white needles: mp 232-233°C; IR (KBr) 3320 [NH], 3301 [NH], 1709 [C=O] cm^-1^; ^1^H NMR (DMSO-*d*_6_) δ 1.32 (t, 3 H, *J =* 4.3 Hz), 2.62 (s, 3 H), 4.29 (q, 2 H, *J =* 4.3 Hz, CH_2_), 7.35 (d, 2 H, *J =* 9.2 Hz, Ar-H), 7.64 (d, 1 H, *J *= 8.3 Hz, Ar-H), 7.67 (d, 1 H, *J *= 8.3 Hz, Ar-H), 7.87 (d, 1 H, *J =* 6.3 Hz, Ar-H), 7.92 (d, 2 H, *J =* 9.2 Hz, Ar-H), 8.15 (d, 1 H, *J =* 6.3 Hz, Ar-H), 8.17 (s, 1 H, Ar-H); 9.08 (s, 1 H, NH), 9.21 (s, 1 H, NH); ^13^C NMR (DMSO-*d*_6_) ppm 14.2 [CH_3_], 24.6 [CH_3_, OCH_2_CH_3_], 60.2 [CH_2_, OCH_2_CH_3_], 113.5-156.6 [Ar-C], 165.4 [C=O]. Anal. (C_20_H_19_NO_3_) C, H, N, S: C, 68.75; H, 5.48; N, 12.03. Found: C, 68.54; H, 5.50; N, 11.91.

#### 1-(2,2,4,4-Tetramethylthiochroman-6-yl)-3-(4-nitrophenyl)urea (10c).

 Amine **14** (221 mg, 1.0 mmol) dissolved in dry THF (6 mL) was cooled to 0°C under N_2_. 4-Nitrophenyl isocyanate (167 mg, 1.02 mmol) in dry THF (5 mL) was added dropwise. The remaining procedure was as for **9a** and gave a brown solid which was subjected to flash chromatography [EtOAc:hexanes, (1:2)] on silica gel. Evaporation of the solvent gave **10c** (257 mg, 72%) as a bright yellow solid: mp 173-174°C; IR (KBr) 3219 [NH], 3189 [NH] cm^-1^; ^1^H NMR (DMSO-*d*_6_) δ 1.33 [s, 6 H, C(CH_3_)_2_], 1.35 [s, 6 H, SC(CH_3_)_2_], 1.90 [s, 2 H, CH_2_], 6.98 [d, 1 H, J = 8.2 Hz, Ar-H], 7.18 [dd, 1 H, J = 8.2, 2.2 Hz, Ar-H], 7.59 [d, 1 H, J = 2.2 Hz, Ar-H], 7.67 [d, 2 H, J = 9.0 Hz, Ar-H], 8.16 [d, 2 H, J = 9.0 Hz, Ar-H], 8.83 [s 1 H, NH], 9.38 [s, 1 H, NH]; ^13^C NMR (DMSO-*d*_6_) ppm 31.2 [C(CH_3_)_2_], 32.1 [SC(CH_3_)_2_], 35.3 [C(CH_3_)_2_], 41.8 [SC(CH_3_)_2_], 53.6 [CH_2_], 117.2-146.4 [Ar-C], 151.90 [C=O]. Anal. (C_20_H_23_N_3_O_3_S) C, H, N, S: calcd, C, 62.32; H, 6.01; N, 10.90; S, 8.32. Found: C, 62.42; H, 6.10; N, 10.74; S, 8.31.

### Experimental Section for X-Ray Analysis

The X-ray intensity data were measured on a Bruker SMART APEX II CCD area detector system equipped with a graphite monochromator and a Mo Kα fine-focus sealed tube (λ = 0.71073 Å) operated at 1.5 kW power (50 kV, 30 mA). The detector was placed at a distance of 6.00 cm from the crystal. The total data collection time was 24 hours for each compound. The frames were integrated with the Bruker SAINT Software [31] package using a narrow-frame integration algorithm. Analysis of the data showed negligible decay during data collection. Data were corrected for absorption effects using the multiscan technique (SADABS) [32]. The structure was solved and refined using the SHELXTL [33] software package. The X-ray structures are in Figs. (**1**,**2**) with hydrogen atoms displayed with an arbitrarily radius.

A clear, light yellow block (0.44 x 0.48 x 0.98 mm) of **10a** was used for the X-ray crystallographic analysis. A total of 2522 frames were collected with a scan width of 0.5° in f and ω with an exposure time of 30 sec/frame. The integration of the data using a monoclinic cell was to 0.80 Å resolution [redundancy 8.2, completeness = 99.6%, R_int_ = 3.77%, R_sig_ = 2.14%]. The final cell constants are based upon the refinement of the XYZ-centroids of 6502 reflections above 2σ (I) with 4.68° < 2θ < 52.74°. The ratio of minimum to maximum apparent transmission was 0.8098. Additional statistics for the X-ray structure determination of **10a** are listed in Table **1**.

A clear, yellow wedge (0.10 x 0.33 x 0.44 mm) of **10c** was used for the X-ray crystallographic analysis. A total of 2519 frames were collected with a scan width of 0.5° in f and ω and an exposure time of 30 sec/frame. The integration of the data using an orthorhombic cell was to 0.80 Å resolution [redundancy 11.3, completeness = 100.0%, R_int_ = 4.06%, R_sig_ = 1.90%]. The final cell constants are based upon the refinement of the XYZ-centroids of 3306 reflections above 2σ (I) with 5.02° < 2θ < 52.74°. The ratio of minimum to maximum apparent transmission was 0.8222. Other information on **10c** is given in Table **1**.

### Biology Methods-Cytotoxicity Assays and Tissue Culture

The CellTiter 96 AQ_ueous_ One Solution Cell Proliferation Assay (Promega) was used to measure living cells remaining after 48 hours of incubation with or without a range of concentrations for the heteroarotinoids. MTS [3-(4,5-dimethylthiazol-2-yl)-5-(3-carboxymethoxyphenyl)-2-(4-sulfophenyl) -2H-tetrazolium, inner salt], a novel tetrazolium compound, was added to each well and incubated for 4 hours, followed by addition of a solublization/stop solution. After overnight incubation, the optical density (OD) of each well was determined by measuring the absorbance at 540 nm using a Dynex MRX Revelation microtiter plate reader. Treatments were performed in triplicate, and the growth indices were derived by dividing the average OD_540_ of each treatment by the average OD_540_ of control cultures. The results represent the average and standard error of two to three independent experiments.

#### Apoptosis Assay.

 Cells were plated in 6-well tissue culture dishes at a cell density of 1 x 10^5^ cells/well. After allowing cells to adhere to the dishes overnight, parallel cultures were treated with varying concentrations of heteroarotinoids or the same volume of DMSO solvent for 24 hours. The Vybrant™ Apoptosis Assay Kit #3 (Molecular Probes) was then utilized to measure apoptosis and necrosis. Tissue culture medium in each well was collected to harvest cells that had already lifted off the tissue culture plate. These were combined with adherent cells that were harvested by trypsinization. The cells were pelleted by centrifugation and resuspended in 100 μL of 1X annexin-binding buffer, and then they were incubated with 5 µl of annexin V conjugated to fluorescein (Annexin-V-FITC) and 1 µL of 100 µg/mL propidium iodide (PI) for 15 minutes at room temperature. The solution was then mixed gently with an additional 400 µl of 1X annexin-binding buffer, and the samples were evaluated with a FACSCalibur (Becton Dickinson) automated benchtop flow cytometer at an excitation wavelength of 488 nm with observation wavelengths of 530 nm and 575 nm. Flow cytometry data were quantified using Summit for MoFlo Acquisition and Sort Control Software (Cytomation, Inc).

#### Tissue Culture.

 The A2780 ovarian cancer cells were cultured in RPMI media, and normal endometrial cells were cultured in MEM media. Both media contained fetal bovine serum (FBS), sodium pyruvate, and antibiotic/antimycotic. The normal endometrial cells were collected from a healthy premenopausal female and used prior to passage [34] as previously described.

## RESULTS AND DISCUSSION

### Chemistry

Key intermediates for the synthesis of members of **9** and **10** were 6-amino-2-methylquinoline (**13**) and 2,2,4,4-tetramethyl-6-aminothiochroman (**14**). Amine **13** is commercial, although only in a purity of 98%, but was used directly without further purification. The thiochroman **14** was not commercial and was prepared [19]. The general methodology is outlined below to obtain **9** and **10**. A critical parameter in the procedure required the use of exceptionally dry THF. Final yields of pure materials were obtained *via *flash chromatography or from one recrystallization of the reaction product from an appropriate solvent. Targets **9** and **10** had sharp melting points, exhibited spectral properties as expected for the structures, were soluble in DMSO (RT), and gave satisfactory elemental analyses. Members of **10** were also soluble in 95% ethanol and absolute ethanol.

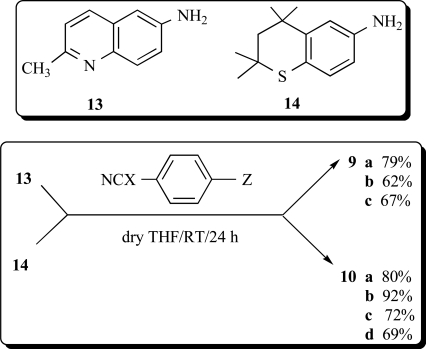



### Structural Analyses of 10a and 10c

The recognition of retinoid-type structures as potential anti-cancer agents for the clinic has received considerable attention [5]. These summaries included a variety of diaryl amides and a small number of *N*,*N*’-diaryl ureas. Recently, several *N*,*N*’-arylalkyl-substituted ureas exhibited other useful biomedical properties [27-29]. In view of the activity found [16-17,19] for certain heteroarotinoids with a urea or thiourea linker between the aryl groups, it was deemed prudent to determine crystal structures of **10a** and **10c** in addition to an examination of ^1^H NMR structural data over a temperature range to ascertain if a certain conformer predominated.

**Fig. (1):**
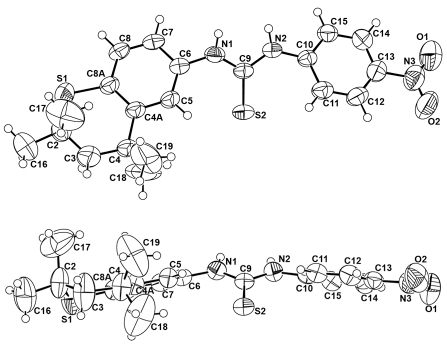
A view of **10a** and **10a** rotated 90° in the Z direction with the numbering scheme employed.

**Fig. (2):**
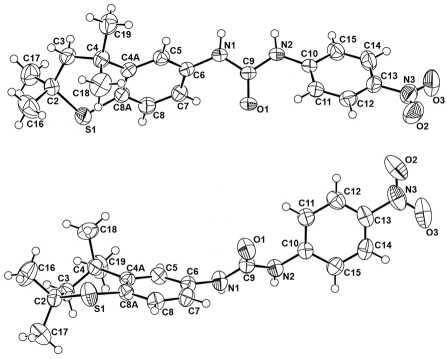
A view of **10c** and **10c** rotated 90° in the Z direction.

The strong activity, the paucity of members of this family of heteroarotinoids, the need for insight as to the favored structure of such systems, and the likely strong H-bonding that could occur prompted an effort to grow crystals of **10a** and **10c** which were the most active. Compounds **10a** and **10c** crystallized as a clear yellow block and a clear yellow wedge, respectively. The crystal and refinement data for both derivatives are presented in Table **1**. Interestingly, **10a** exhibited a monoclinic system while **10c** was orthorhombic. Supplementary information has selected bond distances and bond angles for both crystals. It is clear from the ORTEP drawings (Figs. **1**,**2**) that in the solid state the ring units attached to the two nitrogen atoms in each molecule are in an anti arrangement with respect to each other and in a syn arrangement with respect to the sulfur atom (S2) in **10a** and to the oxygen atom (O1) in **10c**. The entire molecular framework in **10a** is nearly planar while in **10c** the nitro-containing aryl ring is essentially orthogonal to the thiochroman unit. One might expect a “stacking” of rings since the thiochroman unit is electron donating while the nitro-substituted ring is electron withdrawing. Intramolecular ring stacking has been reported for *N*-anisyl-*N*’-(4-nitrophenyl) urea (**15a**) and *N*,*N*’-dimethyl-*N*,*N*’-di(4-nitrophenyl)urea (**15b**) *via *a charge transfer effect/reduced electron repulsiveness between phenyl rings, respectively [35-37]. Indeed, an earlier study revealed that* N*,*N*’-diethyl-*N*,*N*’-diphenylurea crystallized (**15c**) in a syn arrangement in which the two phenyl rings were 3.90 Å apart from center to center. Surprisingly, the center-to-center distance between phenyl rings in *N*,*N*’-dimethyl-*N*,*N*’-di(4-nitrophenyl)urea (**15b**) was even smaller (3.80 Å), presumably due to crowding induced by the two alkyl groups and/or, as suggested by the authors, reduced electron repulsion between the rings. The NCN bond angles were recorded for **15b** and **15c** as 117.8° and 115.2°, respectively, while that for **15a** was not reported. When a large nitro group was placed in an ortho position, the two aryl groups in **16** repositioned anti to each with a NCN bond angle of 115.4° and a distance of 2.11 Å from carbon-to-carbon for the methyl groups [38].

Ring-ring stacking has been postulated for a few ureas as indicated above, in addition to varying syn and anti arrangements for *N*,*N*’-substituted aryl groups [35-40]. Both systems are somewhat flattened in certain regions (Fig. **1**) and have the substituted aryl functions in a syn arrangement to the C=S and C=O bonds, respectively. The torsion angles defined by C6-C5-C4a-C4 in the thiochroman units in **10a** and **10c** are 176.4° and 175.18°, respectively. Thus the thiochroman system deviates slightly from planarity. A striking difference in the two molecules in the solid state is the orthogonal position of the 4-nitrophenyl group in **10c** with respect to the fused, thiochroman ring unit (Fig. **2**). In contrast, the corresponding 4-nitrophenyl group in **10a** is nearly planar with the fused ring counterpart. Moreover, **10a** has a geminal dimethyl group almost facing the C=S bond while in **10c** the geminal dimethyl groups are positioned remotely from the C=O bond, and the entire sulfur-containing portion of the fused ring system is turned away from the C=O group. The NCN’ bond angle was only slightly smaller [112.9°(2)] in **10a** than in **10c** [113.81°(13)]. Interestingly, the corresponding bond angles in thiourea and urea have been reported as 116.0°(±1.0°) and 117.0°(±0.3°), respectively [41-44]. The shorter N2-C9 bond [1.343(4)Å] in **10a**, compared to that in **10c** [1.354(2)Å], may reflect the repulsive nature of the fused ring with the long C=S bond [1.690(3)Å] versus the smaller, highly polarized C=O group [1.2264(19)Å] in **10c**. Interestingly, the N-C bonds in thiourea versus urea have been recorded as 1.33(0.0l)Å and 1.351(0.007)Å, respectively [41]. Surprising was the *para*-substituted phenyl ring in **10c** positioned orthogonal to the fused ring system and away from the C=O bond, presumably to minimize interactions of the latter with the ortho hydrogens of the phenyl ring.

**Fig. (3):**
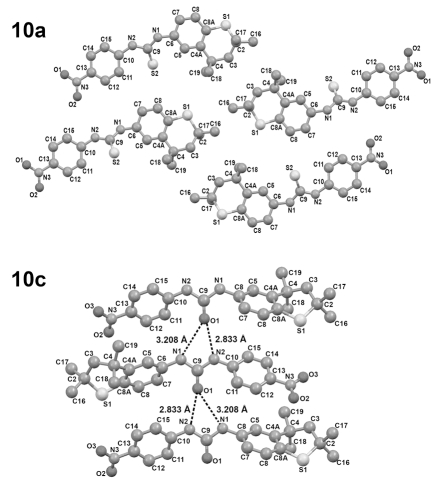
Packing in the crystal structures of **10a** (top) and **10c** (bottom). Important distances for **10a** are N1-S2 = 3.319Å and N2-S2 = 3.370Å [with a molecule in another layer (not shown)] but the hydrogen on the two N atoms are not properly aligned to form good H-bonds to S. In contrast, the corresponding distances for **10c** are N1-O2 = 3.208Å and N2-O2 = 2.833Å, and the hydrogens on the N atoms are aligned well with O and form good H-bonds.

Torsional angles C6-N1-C9-S2 and C6-N1-C9-O1 reflect the slight deviation from planarity of the fused ring with the linking group with values of +1.8(4)° and +6.6(3)°, respectively, in **10a** and **10c**. Similarly, on the opposite side of the C=X bond both torsion angles S2-C9-N2-C10 and O1-C9-N2-C10 possess nearly identical values of -9.5(4)° and -9.8(3)°, respectively, in **10a** and **10c**. In addition, the X-ray determination of single crystals of **10c** demonstrated the existence of a unique lattice network involving hydrogen bonding between hydrogens on N and an oxygen atom of the C=O group in a separate molecule in an *intermolecular* stacked alignment (Fig. **3**) in an alternating “donor-acceptor” type of arrangement. This situation did not persist in **10a** (Fig. **3**), presumably due to a lack of adequate H-bonding between the hydrogens on N with the S atom in a separate molecule. A most interesting feature in the stacked units of **10c** was the alternating positions of the lone phenyl ring above the fused ring unit in individual molecules. The donor-acceptor alignments between molecules must reflect an additional stabilizing property of the lattice in **10c**.

Variable temperature ^1^H NMR studies (400 MHz) of **10a**, **10c**, and **10d** were performed to determine if any major change in conformations occurred over a wide temperature range (Tables **2**,**3**). For the higher temperature study (DMSO-*d*_6_) measurements from 22°C(RT)→40°C→80°C of the chemical shift differences in the two sharp signals for the *two sets* of geminal dimethyl groups revealed ranges from 0.043 ppm to 0.031 ppm for **10a**, 0.017 ppm to 0.010 ppm for **10c**, and 0.018 ppm to 0.010 ppm for **10d**. The implication is clear that very little change occurs in the conformation of the fused ring. The **10a**, **10c**, and **10d** compounds precipitated in DMSO-*d*_6_ around 0°C. Therefore, the chemical shift differences for the same geminal dimethyl groups had to be observed at lower temperatures in DCCl_3_ or acetone-*d*_6_ [22°C(RT)→0°C→-40°C→-80°C] and ranged from 0.048 ppm to 0.059 ppm and 0.046 to 0.081 ppm for **10a** and **10c**, respectively. Solubility limitations prevented accurate measurements at -80°C in both cases. Similar chemical shift measurements involving ester **10d** required acetone-*d*_6_ as the solvent over the same low temperature range and gave differences in the geminal dimethyl signals from 0.010 ppm (80°C) to 0.020 ppm (-80°C). In all of these instances, the small differences reflect very little change in the conformations regarding the fused ring unit containing the partially saturated, flexible thiochroman ring.

An evaluation of the proton signals on the fused aryl group and on the separate aryl group in **10a**, **10c**, and **10d** gave similar results over the temperature range of RT to 80°C in DMSO-*d*_6_ and RT to –40°C in DCCl_3_ as described  above. However, **10d** required acetone-*d*_6_ as the solvent due to solubility limitations in DCCl_3_ for low temperature work. The ^3^J_Ha-Hb_ values for **10a** varied from 9.34 Hz (80°C) to 8.24 Hz (-40°C), again indicating very little change in the conformation. Proton coupling of H_a_-H_b_ in **10c** varied only from 9.34 Hz (80°C) to 8.24 Hz (-40°C). In fact, the ^3^J value was 8.24 Hz at RT as well as at –40°C for **10c** in DCCl_3_. Ester **10d** had ^3^J_Ha-Hb_ values of 8.79 Hz (80°C) to 9.22 Hz (-80°C). Consequently, a reasonable conclusion is that the basic skeletal arrangement in these systems remains nearly constant in solution since all of the differences in ^3^J couplings were less than 1 Hz except for **10a** which had a difference of 1.01 Hz from 80°C to –40°C. In view of the X-ray data, intramolecular H-bonding involving **10c** is defensible from the NMR results.

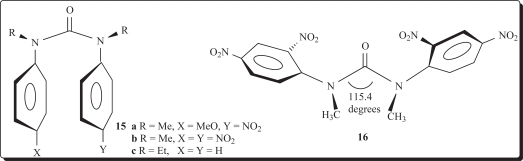



In an overall assessment, one might expect, for example, considerable H-bonding involving the protons on nitrogen in the compounds with DMSO compared to a similar situation in acetone. This should lead to a breakup of any lattice type arrangement, especially in **10c** and to some degree in **10a**. The negligible changes in ^3^J_Ha-Hb_ values for all three compounds implies a type of solvation in both solvents which does not alter the conformation of the systems to any appreciable extent from +80°C to –40°C. In addition, the chemical shifts for the two protons on the para-substituted phenyl ring were essentially unchanged for the three derivatives (δ 7.71-7.85 for H_a_, δ 8.17-8.25 for H_b_-**10a**; δ 7.66-7.32 for H_a_, δ 7.87-8.15 for H_b_-**10c**; δ 7.56-7.81 for H_a_, δ 7.87-8.25 for H_b_-**10d**) which lends credence to our hypothesis that single conformers are present. Moreover, no additional signal splitting was observed at low temperatures which is expected for a single conformer.

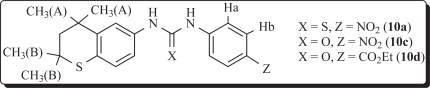



An ^15^N NMR analysis of **10a** and **10c** revealed two signals in each example, namely at 136.78/140.96 ppm for **10a** and 112.02/116.43 ppm for **10c** in DMSO-*d*_6_ at room temperature. Signals for the corresponding ^15^N atom in the respective nitro groups were visible at 372.53 ppm (**10a**) and 372.35 ppm (**10c**). Model systems of substituted thioureas and ureas are rare in the literature [42-43]. Some simple systems with ^15^N signals referenced from NH_3_ (liquid) are as follows: Ph-NO_2_ [^15^N = 370.3 ppm], 4-O_2_N-C_6_H_4_-NH_2_ [^15^N = 371.0 ppm (NO_2_) and 74.5 (NH_2_)], Ph-NH_2_ [^15^N = 59.7 ppm], Ph-NH-C(O)-NH-Ph [^15^N = 108.8 ppm], H_2_N-C(O)-NH_2_ [^15^N = 77.59 ppm], and H_2_N-C(S)-NH_2_ [^15^N = 110.9 ppm]. The marked decreased shielding of ^15^N in thiourea, compared to urea, does not offer an easy explanation since oxygen is more electronegative than sulfur. Tautomerism may exist with some thiourea derivatives which could influence the ^15^N chemical shift [45-47].

### Biological Results and Discussion

The abilities of the new heteroarotinoids to inhibit cancer growth were compared by growing the ovarian cancer cell line (A2780) in the absence and presence of various concentrations of the different compounds. The amount of cells remaining after 48 hours of treatment was measured using a cytotoxicity assay. The optical density (OD) endpoint of the assay represents the number of cells remaining after 48 hours of treatment. The Growth Index was derived by dividing the OD of treated cultures by the OD of untreated cultures. The results are presented in graphic form where Growth Index on the y axis represents the number of cells remaining after 48 hours of treatment relative to untreated controls, and the x axis represents the concentration of drug added. The lower the Growth Index, the greater the growth inhibition.

**Fig. (4):**
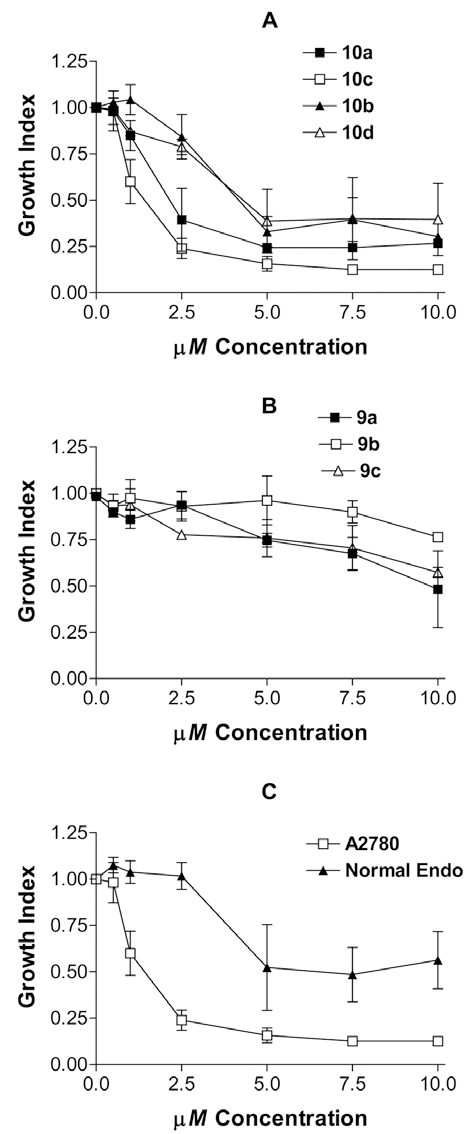
Effects of various concentrations of heteroarotinoids on growth of cancer and normal cells. **A** and **B**) The effect of heteroarotinoids on the number of living A2780 ovarian cancer cells were measured using the Cell Titer 96 Aqueous One Solution Cell Proliferation Assay. The growth index was derived by dividing the optical density corresponding to the number of living cells in the treated culture by the OD of the control cultures treated with solvent only. **C**) The effect of **10c** on retained the differential effect on growth inhibition of cancer versus normal cells (therapeutic window). Direct comparisons of the growth effects of **10a**, **10b**, **10c**, and A2780 ovarian cancer cells and normal endometrial cells (Normal Endo) were compared.

Each of the sulfur-containing heteroarotinoids, **10a**, **10b**, **10c**, and **10d**, inhibited the growth of ovarian cancer cultures in the micromolar range (Fig. **4**). The potencies of the compounds were derived by calculating the EC_50_ values, which are the concentrations that induce half maximal activity (Table **4**). Thus, the lower EC_50_ values represent more potent analogs. In each instance, the analogs with urea linkers were slightly more potent that the corresponding systems with thiourea linkers [Table **4**: compare **10c**-EC_50_ = 1.024 μM, **10a**-EC_50_ = 1.721 μM, **10d**-EC_50_ = 2.814 μM, and **10b**-EC_50_ = 2.935 μM]. Moreover, the agents with the NO_2_ group [**10a**, **10c**] were slightly more potent than the agents with the CO_2_Et group [**10b**, **10d**] (Table **4**). The quinoline derivatives **9a**-**c** exhibited very weak inhibitory activity (Fig. **4**) which indicates that a planar aryl group in that portion of the molecule is ineffective compared to a flexible thiochroman unit present in members of **10**. This is the first recorded observation of this type structure-activity analysis in heteroarotinoids.

Since **10c** exhibited the greatest level of activity, it is important to determine if it retained the differential effect on cancer cells over normal cells and induced apoptosis as previously observed for **10a**, **10b**, and **10d** [17,19]. Fig. (**4C**) and Table **4** show that **10a**, **10b**, **10c** and **10d** induce differential effects on cancer cells versus normal cells demonstrating a significant therapeutic window for the compounds in contrast to that observed with members of **9**. A short term increase in growth index for members of **9** and with **10a** and **10b**, and to a lesser extent the tumor cells (data not included), was not statistically significant.

**Fig. (5):**
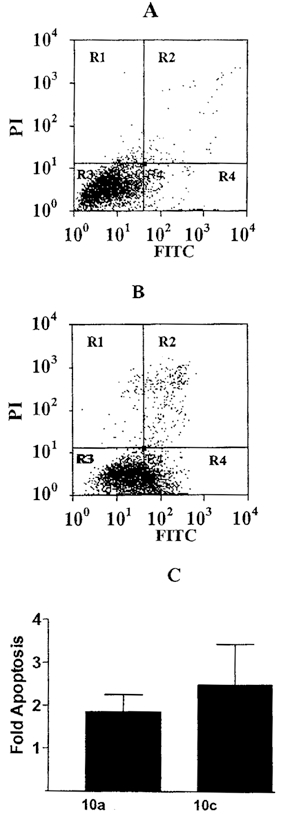
Induction of apoptosis by **10c**. Dual flow cytometric analysis of Annexin-V-FITC and propidium iodide (PI) staining of A2780 cells grown in the absence (**A**) or presence (**B**) of 10 µM **10c**. Living cell populations are clustered in the R3 quadrant, cells in early apoptosis are in the R4 quadrant, and late apoptotic/necrotic cells are in the R2 quadrant. (**C**) The fold induction of apoptosis in cultures was derived by dividing the percentage of cells undergoing apoptosis in treated cultures by the number of cells undergoing apoptosis in untreated control cultures.

To determine if the potent growth inhibition by **10c** is due to induction of apoptosis, A2780 cells treated with 10 µM of the compounds for 24 hours were labeled with Annexin-V-FITC to detect cells undergoing apoptosis and propidium iodide to detect cells undergoing necrosis. Fig. (**5**) demonstrates that **10c** induces positive staining for Annexin-V-FITC in treated cells indicating that apoptosis is occurring. While the majority of cells in the untreated culture (Fig. **5A**) are located in the lower left hand quadrant corresponding to negative staining for FITC and PI and indicating living cells, a significant proportion of the **10c** treated cells have migrated to the lower right hand quadrant (Fig. **5B**) for positive FITC and negative PI indicating cells undergoing apoptosis. Fig. (**5C**) compares the induction of apoptosis by **10c** with **10a** in A2780 cells. Interestingly, the degree of apoptosis induced is slightly higher for **10c** with the urea linker then with **10a** with the thiourea linker.

Fig. (**4B**) demonstrates that **9a** has the highest efficacy and potency, followed by **9c** then **9b** in this experiment. However, it is clear that members of **9** have only a small effect on the growth of cancerous cells and virtually no effect on the growth of normal cells. Consequently, members of **9** did not possess useful anti-cancer activity.

It is important to note that a somewhat related group of heteroarotinoids with semi-flexible linkers (compared to stiff alkenyl and alkynyl linkers between the aryl group) [6] and with a flexible nitrogen-containing tetrahydroquinoline unit and an ester linker illustrated (see below) exhibited modest activity in three ovarian cancer cells lines but was very active against two cancerous vulvar cell lines [14-15]. Such activation by the flexible tetrahydroquinoline unit with an ester linker group, versus ureas or thioureas linkers as in **9** with a planar, nitrogen-containing quinoline ring system, indicates that the heterocyclic ring unit should not be planar but should possess a flexible linker for useful activity.

In another example, the thiochroman-containing system (see below) with the amide linking group exhibited strong inhibition of growth of head and neck squamous cell carcinoma (HNSCC) and induced total remission of tumor growth in 4 of 5 mice [48]. This compound is related to members of **10** and supports the above hypothesis that flexible components in the heteroarotinoid molecule are of significance in inducing useful biological activity.

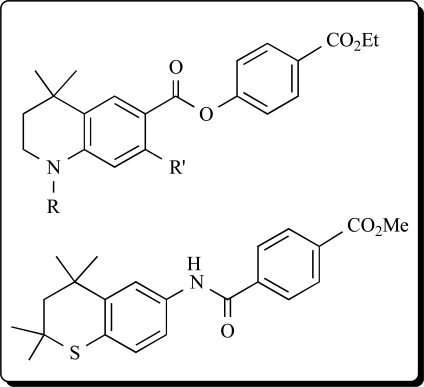



## CONCLUSIONS

In summary, modified heteroarotinoids were constructed with large hydrophobic regions in fused aryl-heterocyclic units. This study evaluated the relationship of structure to anti-cancer activity in terms of a major change in the fused, planar ring unit of substituted quinolines **9** as compared to the flexible thiochroman ring system in members of **10**. This is an important finding to serve as a guideline for the development of more effective heteroarotinoids. Significant toxicity is not expected in these systems, given the lack of toxicity noted with related agents in animal models of cancer [17], skin irritancy [17], and teratogenicity [49]. Preliminary evidence suggests that **10a** can be achieved and maintained in micromolar concentrations in animal models [50] while the mechanism of action to induce apoptosis occurs through direct effects on mitochondria and mitochondrial proteins [25]. Moreover, at 3 μ*M* the effects of **10a** on differentiation has been observed by a normalization of the cancerous cell phenotype and induction of orderly tissue architecture [16].

A range of activities were displayed with all compounds **10** exhibiting inhibition of growth of malignant A2780 ovarian cancer cells with EC_50_ values at micromolar concentrations. Indeed, a good therapeutic window exists between the inhibitory prowess of these agents with the cancer cells versus normal D1 endometrial cells. The data establish that a flexible, thiochroman ring is able to induce apoptosis to a much greater extent than when a planar quinoline unit is present in the heteroarotinoid molecules. Moreover, the orthogonal arrangement of the lone aryl ring in **10c** may be important for induction of apoptosis. Consequently, these small structural changes confirm the subtle characteristics associated in the structure-activity relationships (SAR) of these heteroarotinoids with the A2780 ovarian cancer cell line. Agent **10c** exhibited increased activity, compared to our current lead compound, **10a**, and appears to work through a similar mechanism of action through induction of apoptosis, while retaining a therapeutic window between cancer and normal cells.

A structural investigation of the most active agents **10a**, **10c**, and **10d** using NMR analysis revealed very small changes in selected chemical shifts and in ^3^J_Ha-Hb_ couplings over a wide temperature range. These observations indicate the presence of a single conformation for each compound. In addition, an X-ray determination of a single crystal of **10c** demonstrated the existence of a unique lattice network involving hydrogen bonding between hydrogens on N and an oxygen of the C=O group in a separate molecule in a stacked arrangement (Fig. **3**). This situation did not persist in **10a** (Fig. **3**), possibly due to a lack of H-bonding between the hydrogens on N with the S atom in separate molecules. Another feature in the stacked units of **10c** was the alternating positions of the lone phenyl ring above the fused ring unit in individual molecules.

Since it is known that **10a** binds strongly to proteins in mouse plasma [50], it is possible that **10c** binds to plasma proteins more efficiently than does **10a** because of the strong H-bonding capability of **10c** over **10a** [presence of C=O versus C=S, respectively]. In turn, this could lead to improved binding to potential protein targets by **10c** and therefore potentially enhance efficacy in treating malignant cells. Support for this position is shown in the X-ray analysis which revealed stabilizing intramolecular H-bonding involving the oxygen atom of the carbonyl group in **10c** in contrast to that present in **10a**. Moreover, the NMR data strongly suggested the presence of one conformer for **10c** which is likely due to H-bonding. Compared to the flexible thiochroman unit in members of **10**, the planar quinoline unit in members of **9** was a poor inducer of apoptosis and did not inhibit to any appreciable extent the growth of A2780 ovarian cancer cells. In terms of ability to inhibit cell growth or induce apoptosis, these observations are the first in which a heteroarotinoid system with a flexible, fused ring unit, a flexible linker between aryl groups, and a stabilizing linker have been evaluated with a counter system possessing an aryl unit in a near identical type of molecule. These results will guide construction of future heteroarotinoids for improved activity.

## Figures and Tables

**Table 1:** Crystal and Refinement Data for 10a and 10c

	10a	10c
**Formula**	C_20_H_23_N_3_O_2_S_2_	C_20_H_23_N_3_O_3_S
**FW**	401.53	385.47
**Crystal system**	monoclinic	orthorhombic
**Space group**	P2_1_/c	Pbca
**Crystal size**	0.44x0.48x0.98 mm	0.104x0.329x0.441 mm
**Crystal color**	clear yellow block	clear yellow wedge
**Cell dimensions**	a = 17.4651(18)Å α = 90°	a = 9.4300(19)Å α = 90°
	b = 7.1952(8)Å β = 94.450(7)°	b = 12.980(3)Å β = 90°
	c = 16.2792(17)Å γ = 90°	c = 32.020(6)Å γ = 90°
**Volume**	2039.6(4) Å^3^	3919.3(14) Å^3^
**Density (calcd) g/cm^3^**	1.308	1.307
**Z**	4	8
**Absorption coefficient**	0.281 mm^-1^	0.190 mm^-1^
**Temperature (K)**	298(2)	293(2)
**Radiation**	µ(MoKα)	µ(MoKα)
**Reflections**	33882 measured	45226 measured
	4141 independent	3998 independent
	3283 [(with I>2σ(I)]-observed	3306 [(with I>2σ(I)]-observed
**Diffractometer**	Bruker APEX II CCD	Bruker APEX II CCD
**θ_max_**	26.37° (θ = 2.34-26.37°)	26.37° (θ = 2.51-26.37°)
**Refinement on F^2^**	wR(F^2^) = 0.1947	wR(F^2^) = 0.0815
**w**	1/[σ^2^(F_σ_^2^) + (0.0950P)^2^ + 1.8519P]	1/[σ^2^(F_σ_^2^) + (0.0605P)^2^ + 1.6077P]
	[P = (F_σ_^2^ + 2F_c_^2^)/3]	[P = (F_σ_^2^ + 2F_c_^2^)/3]
**R[F^2^>2σ(F^2^)]**	0.0633	0.0459
**Parameters**	249	249
**(Δ/σ)_max_**	0.580	0.135
**S**	1.037	1.027
**(Δρ)max**	0.844 e/Å^3^	0.500 e/Å^3^
**(Δρ)min**	-0.409 e/Å^3^	-0.368 e/Å^3^
**F(000)**	848	1632

**Table 2:** The Chemical Shift Differences (Δδ) for the Two Sets of Signals for H_3_C(A) and H_3_C(B) for 10a, 10c, and 10d Over the Temperature Range of 80°C to –80°C

Compound	Δδ Value						
Temperature	-80°C	-40°C	0°C	RT	RT	40°C	80°C
**10a** (DCCl_3_)	SL	0.059	0.052	0.048	0.043	0.040	0.031
**10c** (DCCl_3_)	SL	0.081	0.058	0.046	0.017	0.015	0.010
**10d** (Acetone-*d*_6_)	0.020	0.010	NT	0	0.018	0.015	0.010

SL = solubility limitation; NT = not taken; DCCl_3_ freezes at –64°C. ^*^Values were in DMSO-*d*_6_.

**Table 3:** Coupling Constants [^3^J_Ha-Hb_] in Hz and Chemical Shifts for H_a_ and H_b_ for 10a, 10c and 10d from 80°C to –80°C

Compound	10a		10c		10d	
Temp (°C)	^3^J_Ha-Hb_ (Hz)	chem. shift (ppm)	^3^J_Ha-Hb_ (Hz)	chem. shift (ppm)	^3^J_Ha-Hb_ (Hz)	chem. shift (ppm)
80	9.34	H_a_ = 7.85	9.34	H_a_ = 7.66	8.79	H_a_ = 7.56
		H_b_ = 8.17		H_b_ = 8.14		H_b_ = 7.87
40	9.16	H_a_ = 7.82	9.34	H_a_ = 7.66	8.79	H_a_ = 7.56
		H_b_ = 8.19		H_b_ = 8.14		H_b_ = 7.87
RT	9.16	H_a_ = 7.81	9.34	H_a_ = 7.66	8.79	H_a_ = 7.56
		H_b_ = 8.20		H_b_ = 8.15		H_b_ = 7.87
	**DCCl_3_**		**DCCl_3_**		**acetone-*d*_6_**	
RT	9.15	H_a_ = 7.75	8.24	H_a_ = 7.39	9.22	H_a_ = 7.78
		H_b_ = 8.21		H_b_ = 7.94		H_b_ = 8.18
0	8.97	H_a_ = 7.76	8.24	H_a_ = 7.36	NT	NT
		H_b_ = 8.23		H_b_ = 7.92		
-40	8.24	H_a_ = 7.71	8.24	H_a_ = 7.32	9.22	H_a_ = 7.70
		H_b_ = 8.25		H_b_ = 7.87		H_b_ = 8.22
-80	SL	SL	SL	SL	9.22	H_a_ = 7.81 H_b_ = 8.25

Values from RT to 80°C were in DMSO-*d*_6_. The values at the lower temperatures [RT to –80°C] were in DCCl_3_ for **10a** and **10c** and in Acetone-*d*_6_ for **10d**. [SL = solubility limitation; DCCl_3_ freezes at –64°C; NT = not taken].

**Table 4:** Potencies (EC_50_ values) of Growth Inhibition Activity of Cancer Versus Normal Cells

Compound	A2780 Ovarian Cancer	Normal Endometrium
**10a**	1.721 µM ± 0.277	2.969 µM ± 0.501
**10b**	2.935 µM ± 0.163	2.666 µM ± 0.281
**10c**	1.024 µM ± 0.239	2.291 µM ± 0.881
**10d**	2.814 µM ± 0.425	4.536 µM ± 0.945
**9a**	4.998 µM ± 0.495	>1,000 µM
**9b**	7.861 µM ± 0.706	>1,000 µM
**9c**	4.878 µM ± 0.581	>1,000 µM
